# Risk factors to predict drug-resistant pathogens in hemodialysis-associated pneumonia

**DOI:** 10.1186/s12879-016-1701-1

**Published:** 2016-08-08

**Authors:** Ping-huai Wang, Hao-chien Wang

**Affiliations:** 1 Division of Thoracic Medicine, Department of Internal Medicine, Far Eastern Memorial Hospital, New Taipei City, Taiwan; 2 Oriental Institute of Technology, New Taipei City, Taiwan; 3 Division of Thoracic Medicine, Department of Internal Medicine, National Taiwan University Hospital, No. 7, Chung-Shan South Road, Taipei City, Taiwan

**Keywords:** Pneumonia, Hemodialysis, Drug resistant pathogens

## Abstract

**Background:**

After the concept of healthcare associated pneumonia (HCAP) was introduced in 2005 by the American Thoracic Society/Infectious Disease Society of America (ATS/IDSA), pneumonia in hemodialysis patients has been classified as HCAP. Even though there are several risk factors and scoring systems of drug-resistant pathogens (DRPs) in HCAP, the risk factors for DRPs in hemodialysis-associated pneumonia are unclear.

**Methods:**

Patients who were admitted to our tertiary care hospital from January 2005 to December 2010 were screened by a discharge diagnosis of pneumonia. Patients were enrolled if they fulfilled the definition of HCAP according to the 2005 ATS/IDSA guidelines.

**Results:**

A total of 530 subjects were diagnosed with HCAP, of whom 48 (9.1 %) received regular hemodialysis (HD group) and the other 482 did not (non-HD group). The most common pathogens in HD group were *Pseudomonas aeruginosa* and methicillin resistant *Staphylococcus aureus* (MRSA). There was a similar distribution of Gram-negative bacilli infections between the two groups except for *Haemophilus influenzae* and *Citrobacter* species. The incidence of DRPs was not significantly different between the two groups (HD vs. non-HD, 35.4 vs. 39.2 %, *p* = 0.607). Wound care, severe pneumonia and an age of more than 70 years were significant risk factors for DRPs. The area under the operating cure of predicting DRPs was 0.727 (0.575–0.879, *p* = 0.01).

**Conclusion:**

*P. aeruginosa* and MRSA were the most important pathogens in hemodialysis-associated pneumonia. Wound care, severe pneumonia and old age were significant risk factors for DRPs.

## Background

End-stage renal disease (ESRD) has a great impact on global health care. Taiwan had the highest prevalence of ESRD in 2010 according to the United States (US) renal data system 2013 annual report [1], and of these cases, around 90 % underwent hemodialysis [2]. Pneumonia is associated with significant morbidity and mortality in hemodialysis patients. An US study reported that around one third of hemodialysis patients suffered from pneumonia during a 5-year period [3].

The American Thoracic Society/Infectious Disease Society of America (ATS/IDSA) introduced the concept of healthcare-associated pneumonia (HCAP) in 2005, and their guidelines included the risk of drug-resistant pathogens (DRPs) and recommended broad spectrum antibiotics therapy as the treatment of hospital-acquired pneumonia [4]. Hemodialysis patients were close to healthcare facilities. Therefore, according to the 2005 ATS/IDSA guidelines, hemodialysis-associated pneumonia (HDAP) could be considered as a part of HCAP. However, HCAP is a heterogeneous disease entity. Several studies have reported risk factors for DRPs in HCAP, including previous antibiotics exposure, poor activity of daily living or prior residence in a long-term care facility [5]. Although it is clear that hemodialysis patients are at a high risk of blood-stream infections with DRPs [6], the impact of hemodialysis on the risk of DRPs have some arguments. Some studies suggested hemodialysis was one of risk factors for DRPs whereas many others failed to show this association [5, 7, 8].

There might be some risk factors of DRPs specific to patients with hemodialysis. Therefore, we conducted this retrospective study to identify risk factors for DRPs, and to review the demographic and clinical characteristics and microorganisms between HDAP and HCAP.

## Methods

Patients who were admitted to the Far Eastern Memorial Hospital, an 800-bed tertiary care hospital in Taiwan, from January 2005 to December 2010 were screened by the primary discharge diagnosis of pneumonia (International Classification of Diseases codes 482, 485, and 486). The medical records and radiological findings were reviewed to confirm the diagnosis of pneumonia by the following criteria: new or worsening respiratory symptoms; fever, leukocytosis or leucopenia; new or worsening infiltrates on chest plain films pneumonia. Among these pneumonia patients, they were enrolled if they fulfilled the criteria for HCAP, which were defined as follows: patients who had been hospitalized in an acute care hospital for two or more days within the past 90 days; residents of a nursing home or long-term care facility; recipients of recent intravenous antibiotic therapy, chemotherapy or wound care within the past 30 days; or patients who attended a hospital or hemodialysis clinic. The patients who had been transferred in from other hospitals were excluded as their hospital course could not be sure. Demographic, clinical and microbiological data were collected from medical records. The Institutional Review Board of Far Eastern Memorial Hospital approved this study (IRB 102013-E).

A daily steroid dose of more than 10 mg for more than 3 months was defined as steroid use [9]. Chronic kidney disease was defined as an estimated glomerular filtration rate below 30 ml/min without the need for hemodialysis. Active chemotherapy was chemotherapy within the past 60 days for an underlying malignancy. If there were no data of arterial blood gas, oxygen saturation as measured by pulse oximetry (SpO_2_) below 90 % in room air was taken to imply a partial pressure of oxygen below 60 mmHg. Data on causative pathogens were obtained from cultures of respiratory tract secretions such as sputum, tracheal and bronchial aspiration, and/or the cultures of sterile specimens within 72 h of admission including blood or pleural effusion. *Legionella pneumophila* and *Streptococcus pneumoniae* urine antigen tests were also recorded if these exams were checked. The criterion of causative pathogens obtaining from sputum culture was white cell count > 10 per high power field. DRPs were defined as pathogens resistant to community-acquired pneumonia antibiotics regimens such as ampicillin-sulbactam, ceftriaxone, cefotaxime and respiratory quinolone (moxifloxacin or gemifloxacin) In the other words, DRPs included *Pseudomonas aeruginosa* (*P. aeruginosa*), *Acinetobacter* species, *Stenotrophomonas maltophilia* (*S. maltophilia*)*,* methicillin resistant *Staphylococcus aureus* (MRSA), and *Enterobacteriaceae* not sensitive to third generation cephalosporins. The initial antibiotic treatment was classified as being inappropriate if they were not active against the identified pathogens based on in vitro susceptibility testing [10]. β-lactams, quinolones, cephalosporins and carbapenems against *P. aeruginosa*, and anti-MRSA chemotherapy were included as broad-spectrum antibiotics. The pneumonia severity index (PSI) was calculated according to the Pneumonia Patient Outcomes Research Team cohort study for community-acquired pneumonia [11]. Severity was divided into four groups as follows: PSI class II, III, IV, and V as ≤ 70, 71–90, 91–130, > 130, respectively.

All data were expressed as mean ± SD (standard deviation of the mean) unless otherwise stated. Statistical analysis was performed using SPSS version 18 software (SPSS Inc., Chicago, IL, USA). Continuous data were compared using the Student’s *t*-test, and categorical data including demographics, outcomes, antibiotics and microbiology were compared using chi-square distribution (Mann-Whitney test). Multivariate analysis of risk factors was used by general linear model. Comparisons of the clinical characteristics of PSI groups were performed using ANOVA. Significance was taken as *p* < 0.05.

## Results

A total of 530 subjects were diagnosed with HCAP, of whom 48 (9.1 %) received regular hemodialysis therapy (HD group), and the other 482 did not (non-HD group). The clinical characteristics are shown in Table 1. The HD group was significantly younger than the non-HD group (68.3 ± 11.3 vs. 75.8 ± 12.8 years, *p* = 0.001). Pneumonia was less severe in the HD group (*p* = 0.008), and more patients were PSI III but less were PSI IV and V in the HD group. The incidence of diabetes mellitus was higher in the HD group than in the non-HD group (70.8 vs. 38.6 %, *p* < 0.001), however the non-HD group had more comorbidities including cerebrovascular illnesses, malignancy, and chronic obstructive pulmonary disease than the HD group (52.1 vs. 27.1 %, *p* = 0.001; 28.2 vs. 10.4 %, *p* = 0.008; and 37.3 vs. 22.9 %, *p* = 0.047, respectively). Immunosuppression therapy including chemotherapy and steroid therapy were more frequently in the non-HD group (*p* = 0.024 and 0.008).

**Table 1:** Demographic and clinical characteristics

	Total (*N* = 530)	HD(*N* = 48)	Non-HD (*N* = 482)	*p*
Age(years)	75.1 ± 12.8	68.3 ± 11.3*	75.8 ± 12.8	<0.001
Sex(M/F)	349/181	25/23	326/156	
PSI	129.6 ± 33.5	116.8 ± 34.9	130.8 ± 33.2	
PSI group				0.008
PSI II	15(2.8)	4(8.3)	11(2.3)	
PSI III	51(9.6)	11(22.3)*	40(8.3)	
PSI IV	195(36.8)	14(29.2)*	181(37.6)	
PSI V	269(50.8)	19(39.6)*	250(51.9)	
ICU n(%)	116 (21.9)	7(14.6)	109(22.6)	0.2
Admission within 90 d n(%)	317(59.8)	14(29.2)*	303(62.9)	<0.005
Nursing home n(%)	224 (42.3)	10(20.8)*	214(44.4)	0.002
Antibiotics within 90 d n(%)	232 (43.8)	13(27.1)*	219(45.4)	0.015
Active chemotherapy n(%)	47 (8.9)	0*	47(9.8)	0.024
Steroid use n(%)	63(11.9)	0*	63(13.1)	0.008
Wound care n(%)	103 (19.4)	11(22.9)	92(19.1)	0.528
CVA n(%)	264 (49.8)	13(27.1)*	251(52.1)	0.001
Malignancy n(%)	140(26.4)	5(10.4)*	135(28.2)	0.008
DM n(%)	220 (41.5)	34(70.8)*	186(38.6)	<0.005
Heart failure n(%)	84(15.8)	9(18.8)	75(15.6)	0.564
COPD n(%)	191(36)	11(22.9)*	180(37.3)	0.047
Liver cirrhosis	20 (3.8)	0	20(4.1)	0.151

*PSI*, pneumonia severity index, *ICU* intensive care unit, *CVA* cerebrovascular illnesses, *DM* diabetes mellitus, *CKD* chronic renal disease, *COPD* chronic obstructive pulmonary disease, * *p* < 0.05 HD vs. non-HD

Only one subject in the HD group had bacteremia, which was *Klebsiella pneumoniae* (*K. pneumoniae*). The causative microorganisms are shown in Table 2. The yield rate of pathogenic organisms was 43.8 % in the HD group. The incidence of *S. aureus* was similar between the HD and non-HD groups (10.4 vs. 9.3 %). There were also similar distributions of Gram-negative bacilli between the two groups except for *Haemophilus influenzae* (*H. influenzae*) and *Citrobacter* species. None of the patients in the HD group had *H. influenzae*, however 42 patients (8.7 %) in the non-HD group did (*p* = 0.033). *Citrobacter* species were more frequently isolated in the HD group, although the difference was not statistically significant (4.2 vs. 1 %, *p* = 0.07).

**Table 2:** Causative pathogens of hemodialysis-associated pneumonia and non-hemodialysis healthcare-associated pneumonia

	Total (*N* = 530)	HD(*N* = 48)	Non-HD (*N* = 482)
Unknown pathogens n(%)		27(56.2)	217(45.0)
Causative pathogens sensitive to CAP antibiotics regimen			
* S. pneumoniae* n(%)	16 (3.0)	0(0)	16(3.3)
MSSA n(%)	13 (2.5)	0(0)	13(2.7)
β-Streptococcus n(%)	15 (2.8)	2(4.2)	13(2.7)
* K. pneumoniae* n(%)	42(7.9)	2(4.2)	40(8.3)
* E. coli* n(%)	18(3.4)	3(6.3)	23(4.8)
* H. influenzae* n(%)	42(7.9)	0(0)	42(8.7)*
* M.catarrhalis* n(%)	1(0.2)	0(0)	1(0.2)
* M. morganii* n(%)	5(0.9)	0(0)	5(1)
* P. mirabilis* n(%)	23(4.3)	0(0)	23(4.8)
* E. cloacae* n(%)	16(3.0)	3(6.3)	13(2.7)
* S. marcescens* n(%)	33(6.2)	2(4.2)	31(6.4)
Causative pathogens resistant to CAP antibiotics regimen			
MRSA n(%)	37 (7.0)	5(10.4)	32(6.6)
* K. pneumoniae* n(%)	2 (0.4)	0 (0)	2 (0.4)
* E. coli* n(%)	8 (1.5)	0 (0)	0 (0)
* P. mirabilis* n(%)	2 (0.4)	0 (0)	2(0.4)
* P. aeruginosa* n(%)	129(24.3)	8(16.7)	121(25.1)
* A. baumannii* n(%)	25(3.7)	2(4.2)	23(4.7)
* S. maltophila* n(%)	22(4.2)	1(2.1)	21(4.4)
* Citrobacter* species n(%)	7(1.3)	2(4.2)^a^	5(1.0)^b^

*CAP* community acquired pneumonia, *S. pneumoniae Streptococcus pneumoniae, MSSA* methicillin sensitive *Staphylococcus aureus*, *MRSA* methicillin resistant Staphylococcus aureus, K. pneumoniae Klebsiella pneumoniae, E. coli Escherichia coli, H. influenzae Haemophilus influenzae, M. catarrhalis Moraxella catarrhalis, P. mirabilis Proteus mirabilis, E. cloacae Enterobacter cloacae, S. marcescens Serratia marcescens, P. aeruginosa Pseudomonas aeruginosa, A. baumannii Acinetobacter baumannii, S. maltophilia Stenotrophomonas maltophilia.* *p* < 0.05 HD vs. non-HD

^a^ both were *Citrobacter freundii*

^b^ Three were *Citrobacter freundii*; two were *Citrobacter diversus*

**Fig. 1:**
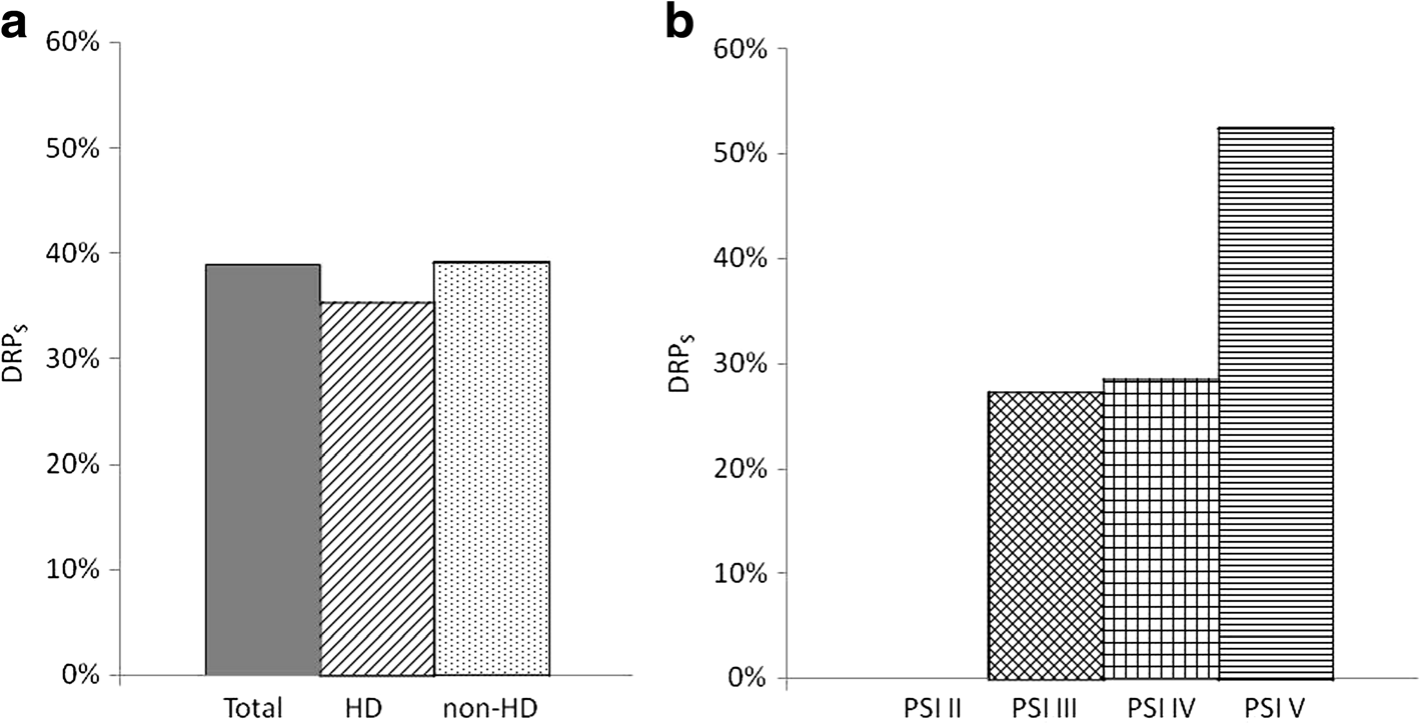
The incidence of drug-resistant pathogens in HDAP, non-hemodialysis HCAP and various PSI groups of HDAP. **a** There was no significant difference in the incidence of DRPs between the HDAP and non-HD HDAP groups. **b** In HD group, there was a trend towards an increasing number of DRPs as the PSI group increased (*p* = 0.16). HDAP: hemodialysis associated pneumonia, HCAP: healthcare associated pneumonia, HD: hemodialysis, DRPs: drug-resistant pathogens, PSI: pneumonia severity index

The incidence of DRPs was not significantly different between the HD and non-HD groups (35.4 vs. 39.2 %, *p* = 0.607). The rates of DRPs stratified by PSI are shown as Fig. 1. In HD group, there were no significant differences in the rate of DRPs among PSI groups, although there was a trend towards an increasing number of DRPs infections as the PSI increased (*p* = 0.16). However, there was a significantly higher risk of DRPs in those with PSI V compared to those with PSV II-IV (*p* = 0.047).

**Fig. 2:**
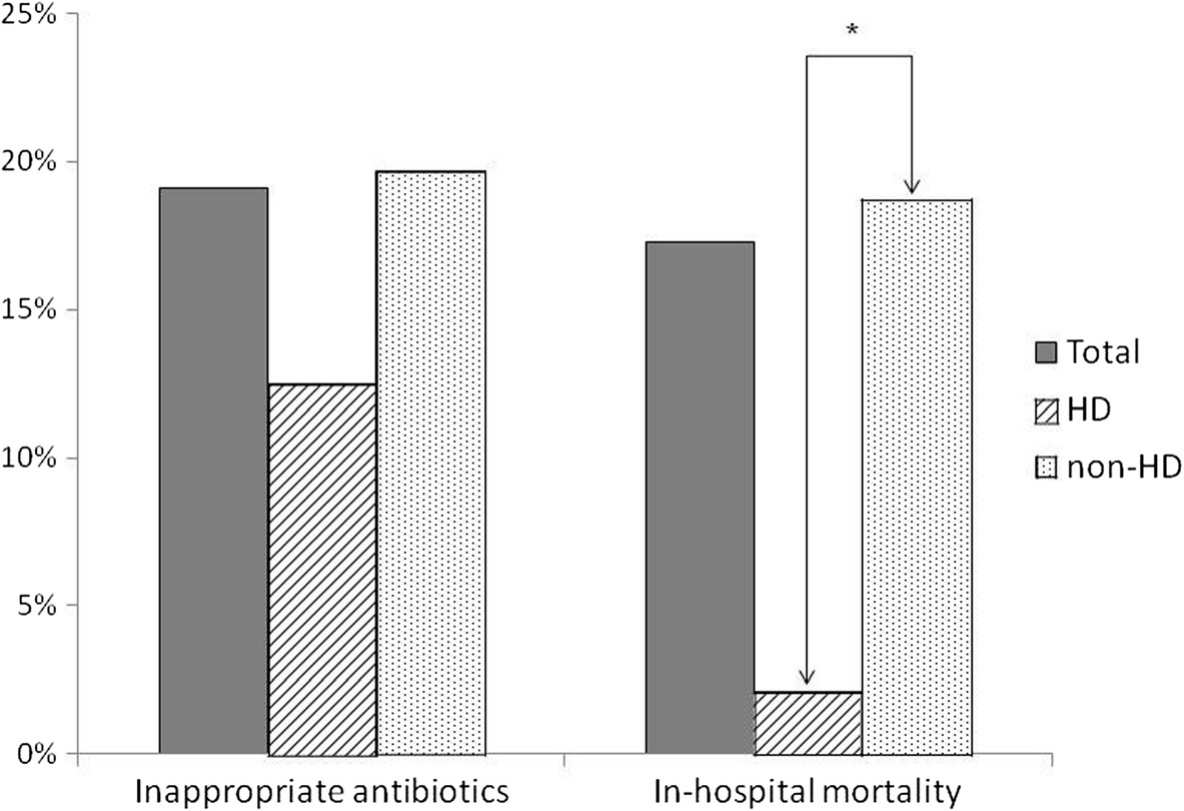
The rate of inappropriate antibiotics and in-hospital mortality rate in patients with HDAP and non-hemodialysis HCAP. There was no significant difference in the rate of inappropriate antibiotic use between the HDAP and non-HD groups. However, the in-hospital mortality rate was lower in the HD group than in the non-HD group (*p* < 0.001). HDAP: hemodialysis associated pneumonia, HCAP: healthcare associated pneumonia, HD: hemodialysis

Shown in Fig. 2, the rate of inappropriate antibiotics use was not significantly different between the HD and non-HD groups (41.7 vs. 46.3 %, *p* = 0.542). However, the in-hospital mortality rate of the HD group was significantly lower than that of the non-HD group (2.1 vs. 18.7 %, *p* = 0.004).

**Fig. 3:**
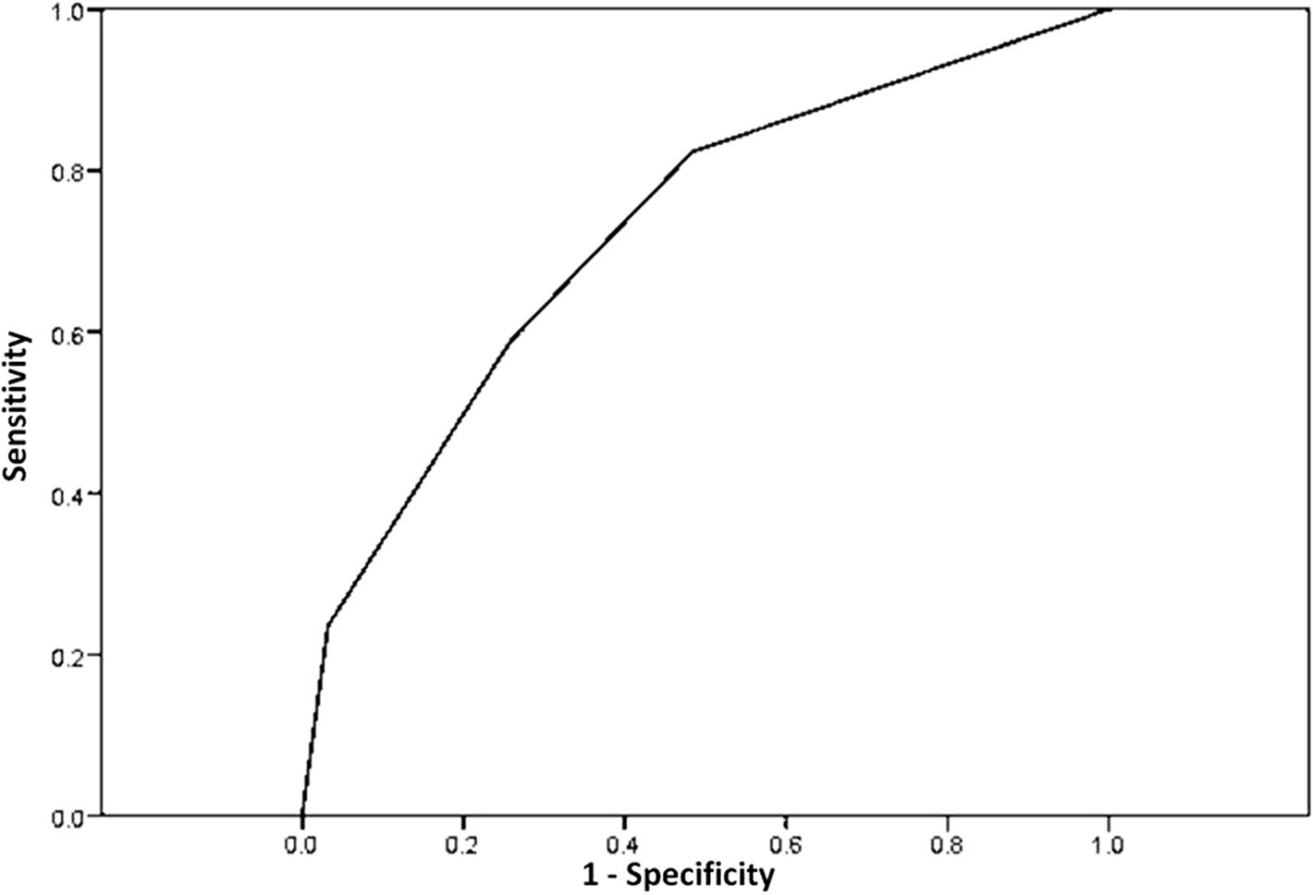
The area under the receiver operating characteristic curve to predict drug-resistant pathogens with the proposed risk factors. The area under the receiver operating curve was 0.727 (0.575–0.879, *p* = 0.01)

Multivariate analysis showed that wound care (OR: 4.73, 95 % of CI: 1.13–19.7, *p* = 0.026), PSI V (OR: 3.49, 95 % of CI: 1.08–12.1) and an age of more than 70 years (OR: 3.81, 95 % of CI: 1.07–13.5, *p* = 0.035) were risk factors for DRPs. Using these three risk factors, the area under the receiver operating curve for predicting DRPs was 0.727 (0.575–0.879, *p* = 0.01) (Fig. 3). In addition, MRSA was an important pathogen in the patients with HDAP who received wound care compared to those who did not receive wound care (36.4 vs. 2.7 %, *p* = 0.001).

## Discussion

The results showed that there were heterogeneous clinical characteristics but similar patterns of pathogens between HD and non-HD groups. The leading pathogens were *P. aeruginosa* and MRSA in the patients with HDAP. The significant risk factors to predict DRPs in the patients with HDAP were wound care, old age (more than 70 years) and PSI V. Wound care not only predicted Gram-negative DRPs but also MRSA.

Even though hemodialysis pneumonia was classified as a sub-type of HCAP in the 2005 ATS/IDSA guidelines, the risk of DRPs in HDAP was not clearly evaluated at that time. Only some indirect evidence supports the role of DRPs and especially MRSA in HDAP [12, 13]. Therefore, Attridge and colleagues suggested that HDAP is considered to be a sub-type of HCAP more by inference than evidence [14]. There is still much debate with regards to HDAP pathogens. A large retrospective studies in the United States (US) showed that the most common Gram-positive bacteria was *S. pneumoniae* [3], even though the role of S. aureus, and especially MRSA is well known in HDAP [15–18]. Furthermore, Pop-Vicas suggested that hemodialysis was not a risk factor for drug-resistant Gram-negative bacteria [19]. In addition, several other studies have suggested the CAP pathogens such as *K. pneumoniae* and *H. influenzae* play important roles in HDAP [3, 15, 16]. *P. aeruginosa* didn’t have the significant impact on HDAP as it suggested in the ATS/IDSA guideline [4]. In contrast, some studies have shown that the DRPs in HDAP are similar to those in nosocomial pneumonia [20, 21]. And the risk of multi-drug resistance has been reported to be higher in patients with HDAP than community-acquired pneumonia pathogens such as *K. pneumoniae* [22]. Our results proved that *P. aeruginosa* and MRSA were the most common pathogens in HDAP, supporting the HCAP concept. The differences in these studies may be associated with different methodologies, geographic factors and local infection control.

PSI was a well-accepted severity scoring system of community acquired pneumonia(CAP) [23]. Khawaja reported that DRPs such as *P. aeruginosa* and *S. aureus* were important pathogens in severe CAP [24]. Falcone and his colleagues proposed that PSI was not as useful in HCAP as in CAP to evaluate outcome and severity [25]. But Falcone reported higher incidence of DRPs in PSI IV and V in community onset pneumonia including HCAP and CAP [26]. Some studies also reported that a critical illness in need of intensive care or mechanic ventilation was a risk factor for DRPs [8, 27, 28]. The similar findings were shown in our study, which showed an upward trend of DRPs incidence as PSI class increased. DRPs incidence in PSI V was significantly higher than PSI II–IV.

Our results showed a lower in-hospital mortality rate in the patients with HDAP than in non-HD patients. The patients with HDAP were younger, had less cerebrovascular disease and shorter stay in long-term care facilities. These characteristics of the patients with HDAP in our study imply a better functional status than non-HD patients. Ewig and colleagues proposed the importance of functional status and daily living activity levels in pneumonia treatment and outcomes [29]. According to PSI, the patients with HDAP had less severe disease. Therefore, it was not surprising that the patients with HDAP had better outcomes.

There are still concerns about which extensive board-spectrum antibiotics should be use for patients with HCAP. Several studies have investigated the risks of DRPs in HCAP with the aim of preventing the overuse of board-spectrum antibiotics. The same issue exists in HDAP. Taylor and colleagues suggested that it was not essential to use antibiotics to cover nosocomial pathogens in every HDAP patient, and that conventional CAP antibiotic therapy may be safe for some hemodialysis patients [17]. However, the inadequate use of empirical antibiotics may increase the mortality rate [30]. Therefore, identifying the risk factors for DPRs is also important in patients with HDAP. Muraya and colleagues reported that age had a marked impact on the prognosis of hemodialysis patients with pneumonia [18]. Our results showed that wound care and severe disease (PSI V) were additional risk factors for DPRs in addition to age. The area under the receiver operating characteristic curve of these three risk factors was 0.727, which is comparable with the studies about risk factors of DRPs in HCAP patients by Shorr and Aliberti [8, 31]. Another reported risk factor associated with DRPs colonization in patients with chronic hemodialysis is antibiotic exposure for more than 7 days in the previous 3 months [19]. The risk factors reported in previous studies did not show a significant correlation with DRPs in the current study. This may be because first-aid in outpatient hemodialysis clinics is readily available, and that partial treatment with oral antibiotics was common before admission.

*Citrobacter* species are commonly found in water, soil and the intestinal tract of human [32]. *Citrobacter* infections usually occur in debilitated, hospitalized patients, with multiple comorbidities [33]. Respiratory tract infections are also common for *Citrobacter* infection [33–35]. We found an incidence of *Citrobacter* species of 4.2 % in HDAP, ranking as the fourth common Gram-negative bacteria pathogens, which was less than that of *P. aeruginosa*, *Escherichia coli* and *Enterobacter cloacae*. To the best of our knowledge, this is the first study to report that *Citrobacter* species are a major HDAP pathogen, even though it had a close relationship with healthcare facilities. This finding may be associated with geographic factors, and further studies are needed to clarify the relationship between *Citrobacter* infection and HDAP.

There were some limitations about this study. It was a single-center retrospective study, and therefore the results may be limited and not generalizable to other regions. The cases number of HD group was small so that it might have negative impact on the power of the study. Furthermore, 56.2 % of HD group and 45 % of non-HD group had no definite pathogens to identify. Although the findings were compatible with clinical experiences and previous studies about pneumonia pathogens, it might limit the interpretation of microbiologic data. It needs a large-scaled prospective study to focus on the epidemiology of HDAP.

## Conclusions

In spite of the small sample size, our results provide additional information with regards to the pathogens than large-scale retrospective studies that did not mostly identify causative pathogens [3, 16]. The reported pathogens in this study confirmed the ATS/IDSA guidelines [4]. The significant risk factors for DRPs in patients with HDAP were old age, wound care and severe pneumonia. Taking these into consideration, the unnecessary use of broad spectrum antibiotics may be avoided.

## Abbreviations

*A. baumannii, Acinetobacter baumannii;* CAP, community acquired pneumonia; CKD, chronic renal disease; COPD, chronic obstructive pulmonary disease; CVA, cerebrovascular illnesses; DM, diabetes mellitus; *E. cloacae, Enterobacter cloacae; E. coli, Escherichia coli;* ESRD, end-stage renal disease; *H. influenzae, Haemophilus influenzae;* HCAP, healthcare associated pneumonia; HD, hemodialysis; HDAP, hemodialysis associated pneumonia; ICU, intensive care unit; *K. pneumoniae, Klebsiella pneumoniae; M. catarrhalis, Moraxella catarrhalis;* MRSA, methicillin resistant *Staphylococcus aureus;* MSSA, methicillin sensitive *Staphylococcus aureus; P. aeruginosa, Pseudomonas aeruginosa; P. mirabilis, Proteus mirabilis;* PSI, pneumonia severity index; *S. maltophilia, Stenotrophomonas maltophilia; S. marcescens, Serratia marcescens; S. pneumoniae, Streptococcus pneumoniae*

## References

[1] Collins AJ, Foley RN, Chavers B, Gilbertson D, Herzog C (2014). US renal data system 2013 annual data report. Am J Kidney Dis.

[2] Wu MS, Wu IW, Shih CP, Hsu KH (2011). Establishing a platform for battling end-stage renal disease and continuing quality improvement in dialysis therapy in Taiwan-Taiwan Renal Registry Data System (TWRDS). Acta Nephrologica.

[3] Slinin Y, Foley RN, Collins AJ (2006). Clinical epidemiology of pneumonia in hemodialysis patients: the USRDS waves 1,3, and 4 study. Kidney Int.

[4] American Thoracic Society, Infectious Disease Scoiety of America (2005). Guidelines for the management of adults with hospital-acquired, ventilator-associated, and healthcare-associated pneumonia. Am J Repir Crit Care Med.

[5] Webb BJ, Dascomb K, Stenehjem E, Dean N (2015). Predicting risk of drug-resistant organisms in pneumonia: moving beyond the HCAP model. Respir Med.

[6] von Baum H, Ober JF, Wendt C, Wenzel RP, Edmond MB (2005). Antibiotic-resistant bloodstream infections in hospitalized patients: specific risk factors in a high-risk population. Infection.

[7] Shorr AF, Zilberberg MD, Reichley R, Kan J, Hoban A (2012). Validation of a clinical score for assessing the risk of resistant pathogens in patients with pneumonia presenting to the emergency department. Clin Infect Dis.

[8] Shorr AF, Zilberberg MD, Micek ST, Kollef MH (2008). Prediction of infection due to antibiotic-resistant bacteria by select risk factors for health care-associated pneumonia. Arch Intern Med.

[9] Stuck AE, Minder CE, Frey FJ (1989). Risk of infectious complications in patients taking glucocorticosteroids. Rev Infect Dis.

[10] Micek ST, Kollef KE, Reichley RM, Roubinian N, Kollef MH (2007). Health care-associated pneumonia and community-acquired pneumonia: a single-center experience. Antimicrob Agents Chemother.

[11] Wu CL, Ku SC, Yang KY, Fang WF, Tu CY (2013). Antimicrobial drug-resistant microbes associated with hospitalized community-acquired and healthcare-associated pneumonia: A multi-center study in Taiwan. J Formos Med Assoc.

[12] Finelli L, Miller JT, Tokars JI, Alter MJ, Arduino MJ (2005). National surveillance of dialysis-associated diseases in the United States, 2002. Semin Dial.

[13] Duran N, Ocak S, Eskiocak AF (2006). *Staphylococcus aureus* nasal carriage among the diabetic and non-diabetic haemodialysis patients. Int J Clin Pract.

[14] Attridge RT, Frei CR (2011). Health care-associated pneumonia: an evidence-based review. Am J Med.

[15] Kawasaki S, Aoki N, Kikuchi H, Nakayama H, Saito N (2011). Clinical and microbiological evaluation of hemodialysis-associated pneumonia (HDAP): should HDAP be included in health-care associated pneumonia?. J Infect Chemother.

[16] Guo H, Liu J, Collins AJ, Foley RN (2008). Pneumonia in incident dialysis patients--the United States Renal Data System. Nephrol Dial Transplant.

[17] Taylor SP, Taylor BT (2013). Health care-associated pneumonia in haemodialysis patients: clinical outcomes in patients treated with narrow versus broad spectrum antibiotic therapy. Respirology.

[18] Muraya Y, Oozono Y, Kadota Y, Miyazaki M, Hashimoto A (1996). Clinical and immunological evaluation of infection in patients on hemodialysis. J Infect Chemother.

[19] Pop-Vicas A, Strom J, Stanley K, D'Agata EM (2008). Multidrug-resistant gram-negative bacteria among patients who require chronic hemodialysis. Clin J Am Soc Nephrol.

[20] Wakino S, Imai E, Yoshioka K, Kamayachi T, Minakuchi H (2009). Clinical importance of Stenotrophomonas maltophilia nosocomial pneumonia due to its high mortality in hemodialysis patients. Ther Apher Dial.

[21] Labelle AJ, Arnold H, Reichley RM, Micek ST, Kollef MH (2010). A comparison of culture-positive and culture-negative health-care-associated pneumonia. Chest.

[22] Lee HL, Whang DH, Park DW, Lee YJ, Kim YH (2013). Higher prevalence of klebsiella pneumoniae extended-spectrum β-lactamase in patients on renal replacement therapy. Korean Med Sci.

[23] Fine MJ, Auble TE, Yealy DM, Hanusa BH, Weissfeld LA (1997). A prediction rule to identify low-risk patients with community-acquired pneumonia. N Engl J Med.

[24] Khawaja A, Zubairi AB, Durrani FK, Zafar A (2013). Etiology and outcome of severe community acquired pneumonia in immunocompetent adults. BMC Infect Dis.

[25] Falcone M, Corrao S, Venditti M, Serra P, Licata G (2011). Performance of PSI, CURB-65, and SCAP scores in predicting the outcome of patients with community-acquired and healthcare-associated pneumonia. Intern Emerg Med.

[26] Falcone M, Russo A, Giannella M, Cangemi R, Scarpellini MG (2015). Individualizing risk of multidrug-resistant pathogens in community-onset pneumonia. PLoS One.

[27] Brito V, Niederman MS (2009). Healthcare-associated pneumonia is a heterogeneous disease, and all patients do not need the same broad-spectrum antibiotic therapy as complex nosocomial pneumonia. Curr Opin Infect Dis.

[28] Maruyama T, Fujisawa T, Okuno M, Toyoshima H, Tsutsui K (2013). A new strategy for healthcare-associated pneumonia: a 2-year prospective multicenter cohort study using risk factors for multidrug-resistant pathogens to select initial empiric therapy. Clin Infect Dis.

[29] Ewig S, Welte T, Chastre J, Torres A (2010). Rethinking the concepts of community-acquired and health-care-associated pneumonia. Lancet Infect Dis.

[30] Zilberberg MD, Shorr AF, Micek ST, Mody SH, Kollef MH (2008). Antimicrobial therapy escalation and hospital mortality among patients with health-care-associated pneumonia: a single-center experience. Chest.

[31] Aliberti S, Di Pasquale M, Zanaboni AM, Cosentini R, Brambilla AM (2012). Stratifying risk factors for multidrug-resistant pathogens in hospitalized patients coming from the community with pneumonia. Clin Infect Dis.

[32] Lipsky BA, Hook EW, Smith AA, Plorde JJ (1980). Citrobacter infections in humans: experience at the Seattle Veterans Administration Medical Center and a review of the literature. Rev Infect Dis.

[33] Samonis G, Karageorgopoulos DE, Kofteridis DP, Matthaiou DK, Sidiropoulou V (2009). Citrobacter infections in a general hospital: characteristics and outcomes. Eur J Clin Microbiol Infect Dis.

[34] Shih CC, Chen YC, Chang SC, Luh KT, Hsieh WC (1996). Bacteremia due to citrobacter species: significance of primary intraabdominal infection. Clin Infect Dis.

[35] Mohanty S, Singhal R, Sood S, Dhawan B, Kapil A (2007). Citrobacter infections in a tertiary care hospital in Northern India. J Infect.

